# Induction, Proliferation, Regeneration and Kinsenoside and Flavonoid Content Analysis of the *Anoectochilus roxburghii* (Wall.) Lindl Protocorm-like Body

**DOI:** 10.3390/plants11192465

**Published:** 2022-09-21

**Authors:** Hongzhen Wang, Xinying Chen, Xiaoyun Yan, Zhixia Xu, Qingsong Shao, Xueqian Wu, Lingjuan Tou, Li Fang, Mingming Wei, Huasen Wang

**Affiliations:** 1 State Key Laboratory of Subtropical Silviculture, Zhejiang Provincial Key Laboratory of Resources Protection and Innovation of Traditional Chinese Medicine, Department of Chinese Herbal Medicine Zhejiang Agriculture and Forestry University, Hangzhou 311300, China; 2 Key Laboratory for Quality and Safety Control of Subtropical Fruits and Vegetables, Collaborative Innovation Center for Efficient and Green Production of Agriculture in Mountainous Areas of Zhejiang Province, Ministry of Agriculture and Rural Affairs, College of Horticulture Science, Zhejiang Agriculture and Forestry University, Hangzhou 311300, China; 3 Jinhua Academy of Agricultural Sciences, Jinhua 321017, China; 4 Ministry of Agriculture Key Laboratory of Biology and Genetic Resource Utilization of Rubber Tree, State Key Laboratory Breeding Base of Cultivation and Physiology for Tropical Crops, Rubber Research Institute, Chinese Academy of Tropical Agricultural Sciences, Danzhou 571700, China

**Keywords:** *Anoectochilus* *roxburghii* (Wall.) Lindl, IPR-PLB, propagation, kinsenoside, flavonoids

## Abstract

*Anoectochilus roxburghii* (Wall.) Lindl has been used in Chinese herbal medicine for treating various ailments. However, its wild resources are endangered, and artificial cultivation of the plant is limited by the low regeneration rate of conventional propagation methods. The lack of *A. roxburghii* resources is detrimental to the commercial production of the plant and kinsenoside, which is unique to *Anoectochilus* species. To develop highly efficient methods for *A. roxburghii* micropropagation and find alternative resources for kinsenoside production, we created an induction, proliferation, and regeneration of PLBs (IPR-PLB) protocol for *A. roxburghii*. We also analyzed the kinsenoside and flavonoid contents during the induction and proliferation of PLBs. The best media of IPR-PLB for PLB induction and proliferation (secondary PLB induction and proliferation), shoot formation, and rooting medium were Murashige and Skoog (MS) + 3 mg/L 6-benzylaminopurine (6-BA) + 0.5 mg/L naphthaleneacetic acid (NAA) + 0.8 mg/L zeatin (ZT) + 0.2 mg/L 2,4-dichlorophenoxyacetic acid (2, 4-D), MS + 3 mg/L 6-BA + 0.5 mg/L NAA, and MS + 0.5 mg/L NAA, respectively. On these optimized media, the PLB induction rate was 89 ± 2.08%, secondary PLB induction rate was 120 ± 5%, secondary PLB proliferation rate was 400 ± 10% and 350 ± 10 % in terms of the quantity and biomass at approximately 1 month, shoot induction rate was 10.5 shoots/PLB mass, and root induction rate was 98%. All plantlets survived after acclimation. Darkness or weak light were essential for PLB proliferation, and light was crucial for PLB differentiation on these optimized media. The kinsenoside contents of PLBs and secondary PLBs were 10.38 ± 0.08 and 12.30 ± 0.08 mg/g fresh weight (FW), respectively. Moreover, the peak kinsenoside content during the proliferation of secondary PLBs was 34.27 ± 0.79 mg/g FW, which was slightly lower than that of the whole plant (38.68 ± 3.12 mg/g FW). Two flavonoids exhibited tissue- or temporal-specific accumulation patterns, and astragalin accumulated exclusively during the first 2 weeks of cultivation. The IPR-PLB protocol for *A. roxburghii* may facilitate the efficient micropropagation of *A. roxburghii* plants. Furthermore, the PLBs are a good alternative resource for kinsenoside production.

## 1. Introduction

*Anoectochilus roxburghii* (Wall.) Lindl is one of the main medicinal plants in the family of Orchidaceae. It is used in Chinese herbal medicine for treating diabetes, hyperlipidemia, liver disease, lung disease, snake bites, and cancer [1,2,3]. It is called the “king of medicine” because of its diverse pharmacological effects [4], and it is mainly distributed in the tropical and subtropical regions of China (e.g., Fujian, Zhejiang, and Guangxi), and sparsely distributed in Japan, Vietnam, and Thailand [5]. Its wild resources are under the threat of extinction because of the loss of suitable habitats and the excessive collection by humans. Accordingly, it has been listed as a vulnerable, near-threatened, or endangered species by the Convention on International Trade in Endangered Species of Wild Fauna and Flora (https://cites.org/eng/disc/text.php (accessed on 20 March 2022)) and as a second-class protected plant in the Chinese National Wild Conservative Plants List (http://www.plant.csdb.cn/protectlist (accessed on 20 March 2022)). Its market supply mainly depends on the artificial cultivation of propagated plants. Clonal multiplication by induction of shoots from stem nodes is a main method for propagating *A. roxburghii* [6,7]; however, the limited efficiency of this method has hindered the large-scale cultivation of clonal plants [8]. The lack of *A. roxburghii* resources has resulted in very high prices of this plant on the market, which are CNY 600–1000/kg fresh weight (FW) and CNY 5000–10,000/kg dry weight (DW). Hence, there is an urgent need for studies on improving *A. roxburghii* clonal propagation.

The orchid protocorm-like body (PLB) is a somatic embryo. Previous research indicated somatic embryogenesis and PLB formation refer to the same developmental event [9,10]. The general growth and structural characteristics of orchid PLBs are similar to those of protocorms [9,10]. The main difference between clonal propagation and sexual reproduction, which involves fertilization, zygotic embryogenesis, and the germination and formation of protocorms, is that PLBs derive from somatic tissues, therefore being considered a type of vegetative propagation [10]. The PLB has mainly been reported for orchid plants and rarely in non-orchid plants [11,12,13]. It can divide, proliferate, and merge continuously to form secondary PLB masses [14,15,16,17,18]. This process is also called PLB proliferation, which involves secondary PLB induction and proliferation [14,15,16,17,18]. Orchid PLBs have a very high regenerative potential [14], making the induction, proliferation, and regeneration of PLBs (IPR-PLB) an ideal strategy for the mass propagation of orchids [10]. A major advantage of this strategy over conventional clonal propagation is its increased multiplication rate [10,14]. Thus, the utility of IPR-PLB has been investigated for many orchid plants, including *Cyrtopodium paludicolum* [19], *Grammatophyllum speciosum* [20], *Coelogyne cristata* and *Coelogyne flaccida* [21,22], and *Phalaenopsis* and *Oncidium* hybrids [10]. However, IPR-PLB responses are highly dependent on the genotype, which is a substantial obstacle to using an established orchid IPR-PLB protocol for the clonal propagation of another orchid PLB [10]. Studies on *A. roxburghii* PLB have mostly focused on the induction and proliferation of PLBs [15,16,17,18]. There is currently a lack of research on the IPR-PLB of *A. roxburghii*. Therefore, a systematic investigation of IPR-PLB protocols of *A. roxburghii* is needed. In addition to their utility for micropropagations, PLBs are also an attractive alternative for the production of useful plant-specific metabolites. For example, the *Dendrobium* Sabin Blue PLBs may be used as a source of dendrobine [23].

**Figure 1:**
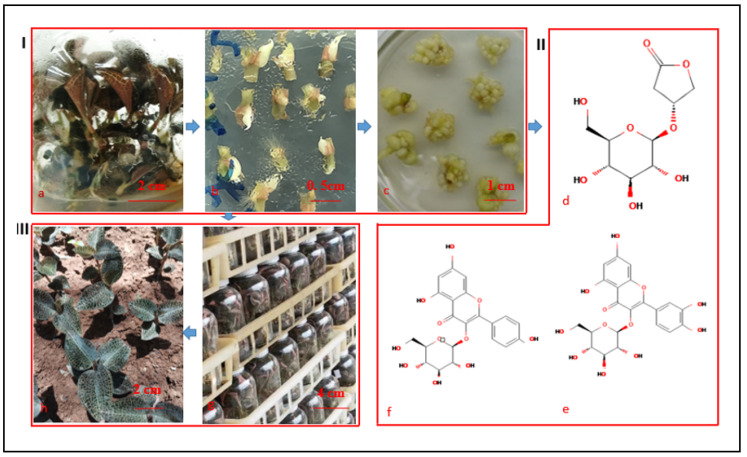
Flowchart of this study. (**I**) PLB induction and collection. (**II**) Bioactive components. (**III**) Mass propagation, acclimation, and artificial cultivation of *A. roxburghii* plant. (**a**) Mother plants. (**b**) PLB induction. (**c**) PLB proliferation (secondary PLB induction and proliferation). (**d**) Kinsenoside (PubChem CID: 10422896). (**e**) Isoquercitrin (PubChem CID: 5280804). (**f**) Astragalin (PubChem CID: 5282102). (**g**) PLB regeneration. (**h**) Artificial cultivation of propagated plants which have been acclimated.

Kinsenoside (Figure 1d), which is a unique compound produced by *Anoectochilus* plants [3,24,25], has considerable antihyperglycemic, antihyperliposis, and hepatoprotective activities, as well as antiosteoporotic, anti-inflammatory, and vascular protective effects under high-glucose conditions [3,24,25]. The chemical synthesis of kinsenoside is seriously limited by high costs and low yields [24]. Moreover, the large-scale production of kinsenoside from *Anoectochilus* plants is not feasible because of the relative lack of natural resources. Thus, kinsenoside is supplied commercially only in trace amounts at extremely high prices of CNY 800–1000/10 mg. The exploitation of *A. roxburghii* PLBs may be a viable alternative used for the commercial production of kinsenoside. Flavonoids, which are the main bioactive compounds in *A. roxburghii* plants [3], have hepatoprotective effects and antitumor and antioxidant activities [3]. However, there are no reports describing the content and distribution of kinsenoside and flavonoid monomers, such as isoquercitrin (Figure 1e) and astragalin (Figure 1f), in *A. roxburghii* PLBs.

Although earlier studies have generated valuable data regarding the IPR-PLB of orchids, there are no protocols or cultivation conditions that are appropriate for all genotypes because of the diversity in genomic sequences, explants, hormones, and required culture conditions. Therefore, an efficient IPR-PLB protocol applicable for A. roxburghii must be developed and the kinsenoside and flavonoid contents in PLBs should be analyzed. In this study, PLBs were induced from stem nodes and converted into well-developed plantlets via the sequential shoot and root formation in vitro. The plantlets were subsequently acclimatized under optimized ex vitro conditions. Additionally, the kinsenoside and flavonoid contents in the induced and proliferated PLBs were examined. A flowchart summarizing this study is presented in Figure 1.

## 2. Results

### 2.1. Induction of PLBs

**Figure 2:**
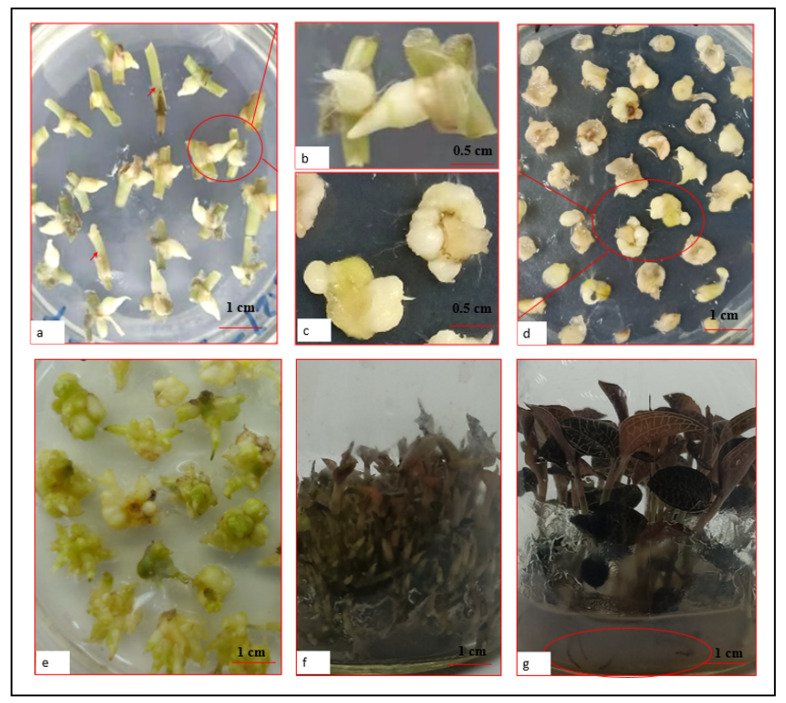
Induction, proliferation, and regeneration of *A. roxburghii* PLB. (**a**) Induction of *A. roxburghii* PLBs. The arrows indicate stem nodes near apical shoot. (**b**) Magnification (4×) of the selected area in panel a. (**c**) Magnification (4×) of the selected area in panel d. (**d**) Secondary PLB induction. (**e**) Mastoid PLB mass. (**f**) Shoot formation. (**g**) Root formation (the roots are within the circled region).

Stem segments with nodes were placed on solid Murashige and Skoog (MS) medium with or without hormones (Table 1) for the induction of PLBs (Figure 2a) at 25 ± 2 °C with a 16 h light/8 h dark photoperiod (1500–2000 lux irradiation). The addition of hormones decreased time taken for PLB occurrence and increasedPLB induction rate. Compared with MS devoid of hormones, the addition of 6-benzylaminopurine (6-BA), naphthaleneacetic acid (NAA), and zeatin (ZT) decreased the time required for PLBs occurring and increased the induction rate by 5 days and 5% and 4 days and 20% on MS + 3 mg/L 6-BA + 0.5 mg/L NAA and MS + 3 mg/L 6-BA + 0.5 mg/L NAA + 0.8 mg/L ZT, respectively (Table 1). Compared with MS + 3 mg/L 6-BA + 0.5 mg/L NAA + 0.2 mg/L ZT, the addition of ZT at 0.8 mg/l decreased the occurrence time of PLBs and enhanced the PLB induction rate slightly (Table 1). The PLB appearance time was shortest (19 days) and PLB induction rate was highest (89 ± 2.08%) on MS + 3 mg/L 6-BA + 0.5 mg/L NAA + 0.8 mg/L ZT + 0.2 mg/L 2, 4-D. The PLB induction rate was considerably higher for the nodes in the middle of the stem than for the nodes at the stem base. Moreover, PLBs were not induced from the nodes near apical shoots (Figure 2a). The PLBs were initially detected at the nodes as milky white or white globular protuberances at approximately 19 days post-inoculation (dpi). Most of these protuberances formed white spiky PLBs at approximately 30 dpi on the optimized medium (Figure 2a,b), although there were a few elliptical, short rod-like, or irregularly shaped PLBs.

### 2.2. Proliferation of PLBs (Induction and Proliferation of Secondary PLBs)

**Figure 3:**
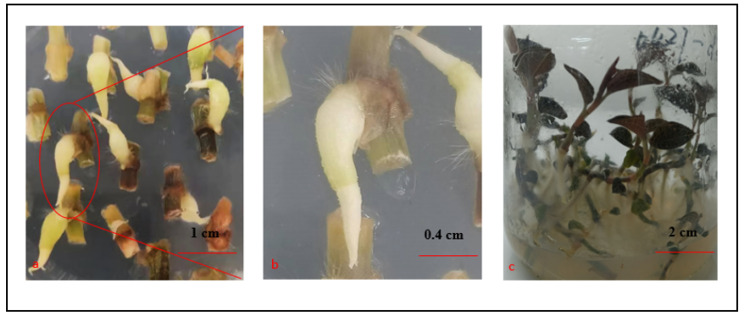
Differentiation of the PLBs incubated with a 16 h light/8 h dark photoperiod (1500–2000 lux irradiation). (**a**) PLBs after a 15-day incubation. (**b**) Magnification (3×) of the selected area in panel a. (**c**) PLBs differentiated into plantlets.

The PLBs cultivated for 15 days with a 16 h light/8 h dark photoperiod (1500–2000 lux irradiation) turned light or dark green and differentiated to form leaf primordia (Figure 3a,b). The cultivation under the same conditions for 45 days resulted in the further differentiation of the PLBs with leaf primordia and the formation of leaves and roots (Figure 3c). Accordingly, light induced the differentiation, but not the proliferation, of the PLBs. Similar to the conventional clonal propagation of *A. roxburghii* from stem nodes, one plantlet was regenerated from one PLB (Figure 3c).

Transverse sections (2–3 mm thick) of the PLBs at 30–35 dpi were cut and added to the optimized medium, which was then incubated in darkness or under weak light. The PLBs divided, proliferated, and merged into secondary cauliflower-shaped PLB masses at 30 dpi (Figure 2c,d). These secondary PLBs germinated to produce new PLBs at 30–60 dpi (Figure 2e). The secondary PLBs merged and formed loose mastoid PLB masses at around 60 dpi (Figure 2e), with an induction rate of 120 ± 5%. Almost all of the basal PLB sections produced secondary PLB masses, whereas only a few apical PLB sections did. The basal PLB sections had a higher induction rate than the apical sections. Intact PLBs that were selected at 30–35 dpi and then incubated in darkness or under weak light also produced loose mastoid PLB masses (Figure 2e) after 60 dpi.

**Figure 4:**
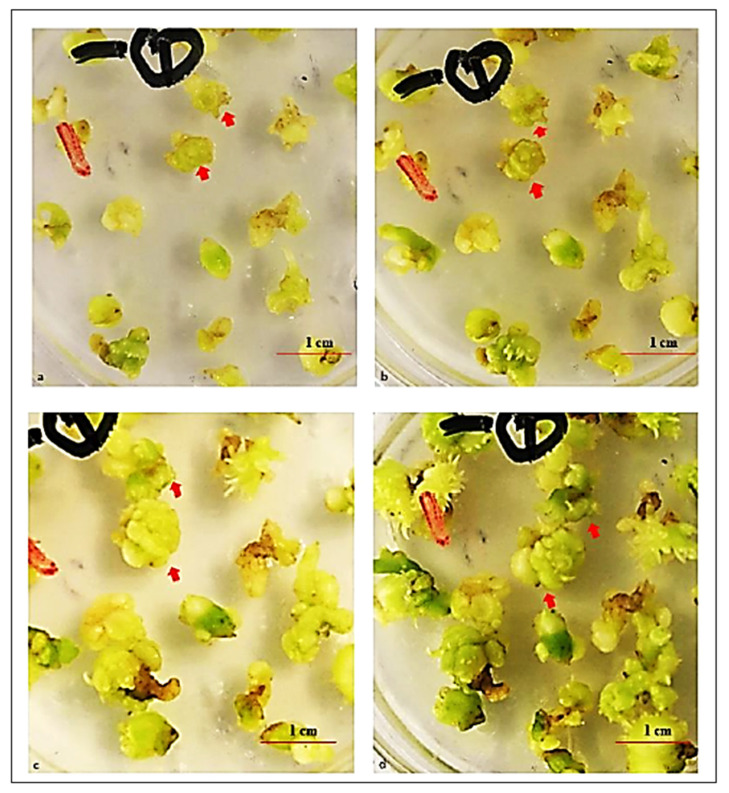
Secondary PLB proliferation after culturing for (**a**) 1 week, (**b**) 2 weeks, (**c**) 4 weeks, and (**d**) 5 weeks. The arrows indicate PLBs with a defined shape.

Transverse sections of the mastoid PLB masses were cut and inoculated on the optimized medium, which was then incubated in darkness or under weak light for the proliferation of PLBs. A few secondary PLBs germinated from the edge of the cut sections and merged into a tight cauliflower-like mass after a 2-week cultivation (Figure 4a,b). These PLBs generated more new PLBs, and merged into a tight cauliflower-like mass after a 4-week cultivation. The surface of the resulting mass was covered with many PLBs (Figure 4c,d). No PLBs germinated, but the secondary PLBs differentiated and formed many spines after a 5-week cultivation under weak light (Figure 4d). Moreover, the PLBs continuously expanded during a 5-week cultivation in darkness. The secondary PLB proliferation rates in terms of quantity and biomass were 400 ± 10% and 350 ± 10%.

### 2.3. Kinsenoside and Flavonoid Contents of PLBs

**Figure 5:**
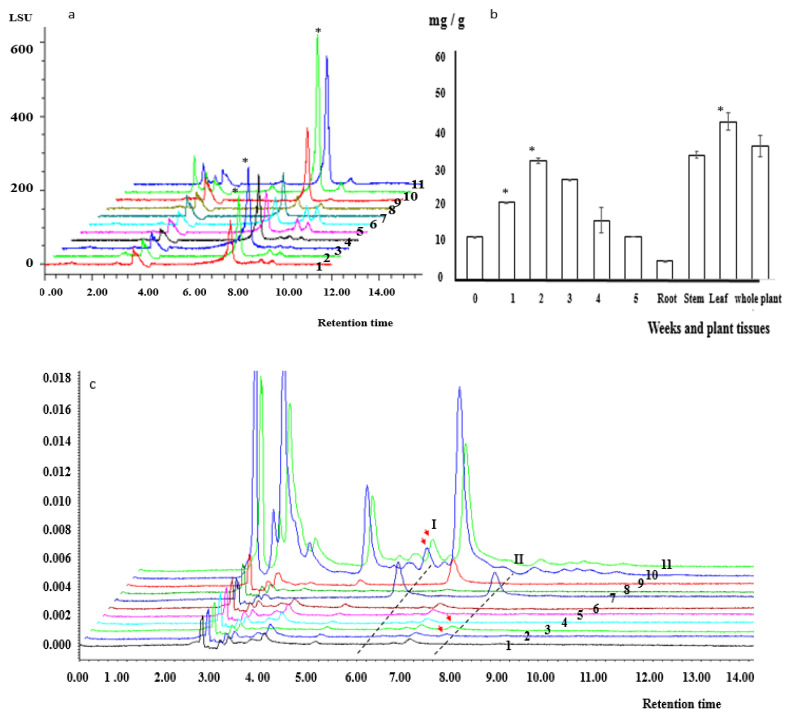
Kinsenoside, isoquercitrin, and astragalin contents in *A. roxburghii* PLBs and plants. (**a**) HPLC chromatograms of kinsenoside contents; 1–6 correspond to 0–5 weeks of culturing, respectively, 7 corresponds to the kinsenoside standard, and 8–11 correspond to the roots, stem, leaf, and whole plant, respectively. The asterisk indicates the kinsenoside content was significantly different from that of roots (*p* < 0.05). (**b**) Kinsenoside contents in PLBs and plant tissues; the numbers on the x-axis refer to the number of weeks. (**c**) Isoquercitrin (I) and astragalin (II) contents in different tissues of *A. roxburghii* plants; 1–6 correspond to 0–5 weeks of culturing, respectively, 7 corresponds to the isoquercitrin (I) and astragalin (II) standards, and 8–11 correspond to the root, stem, leaf, and whole plant, respectively. Arrows indicate the peak isoquercitrin and astragalin levels.

The changes in kinsenoside and flavonoid contents were monitored during the induction and proliferation of PLBs as well as in different tissues of the whole plant. The kinsenoside content was analyzed using the HPLC–evaporative light-scattering detector (ELSD). On the basis of its major peak in the HPLC chromatograms, kinsenoside had a retention time of 7.77 min. The kinsenoside content in PLBs, secondary PLBs, roots, stems, leaves, and whole plants were 10.38 ± 0.08, 12.30 ± 0.08, 5.39 ± 0.22, 35.98 ± 1.02, 45.61 ± 2.39, and 38.68 ± 3.12 FW, respectively. The kinsenoside content during the proliferation of the secondary PLBs increased and peaked (34.27 ± 0.79 mg/g FW) at 2 weeks, after which it decreased gradually to the initial level (i.e., 0-week time-point) (Figure 5a,b). The highest kinsenoside content in the proliferating secondary PLBs was slightly lower than the kinsenoside content of the whole plant (Figure 5a,b).

Isoquercitrin and astragalin contents were determined using the HPLC-PDA system. The major isoquercitrin and astragalin peaks in the HPLC chromatograms were at 5.77 and 7.77 min, respectively (Figure 5c). Trace amounts of isoquercitrin accumulated in the leaves (0.36 ± 0.005 mg/g FW) and in the whole plant (0.35 ± 0.005 mg/g FW) (Figure 5c). Astragalin accumulated only during the first 2 weeks of the cultivation period, and its contents were 0.20 ± 0.007 and 0.35 ± 0.0006 mg/g FW in weeks 1 and 2, respectively (Figure 5c).

### 2.4. Regeneration of PLBs and the Formation of Shoots and Roots

The proliferated PLB masses were used to inoculate the solid shoot induction medium (Table 2), which was then incubated at 25 ± 2 °C with a 16 h light/8 h dark photoperiod (1500–2000 lux irradiation) for shoot formation. The shoot induction rate and shoot diameter at 60 dpi were greater for the MS + 6-BA + NAA than those for the MS medium lacking hormones (Table 2), with the exception of the MS medium containing 5 mg/L 6-BA, which had a lower shoot induction rate than the MS medium lacking 6-BA (Table 2). Shoots were induced on the medium without hormones, but the induction rate and shoot diameter were 5.0 shoots/PLB mass and 2.02 ± 0.44 mm, respectively (Table 2). The shoot induction rate increased in response to an increase in the 6-BA/NAA ratio, peaking at 10.5 shoots/PLB mass, with a shoot diameter of 3.19 mm, on the MS + 3 mg/L 6-BA + and 0.5 mg/L NAA (Table 2). Further increases in the 6-BA concentration adversely affected shoot formation (Table 2). The lowest number of shoots (4.3 shoots/PLB mass) was recorded for the MS + 5 mg/L 6-BA and 0.5 mg/L NAA (Table 2). The addition of 6-BA and NAA also influenced the shoot length, which decreased as the 6-BA concentration increased. The shortest shoots (2.43 mm) were detected on MS + 5 mg/L 6-BA + 0.5 mg/L NAA (Table 2).

Individual shoots (1.5–2 cm in height) were transferred to rooting medium (Table 3), which was then incubated at 25 ± 2 °C with a 16 h light/8 h dark photoperiod (1500–2000 lux irradiation) to induce root formation. After a 10-day incubation, 2–4 roots were detected near the incision site. Additionally, the roots were covered with white root hairs. At 30 dpi, the MS + 0.5 mg/L NAA had the highest rooting rate (98.35 ± 0.05%), the highest number of roots (3.22 ± 0.21), and the longest roots (3.51 ± 0.12 cm) (Table 3).

### 2.5. Acclimation of In Vitro Grown Plantlets

Fully developed plantlets (3–4 cm) with well-developed roots were acclimatized for about 2 weeks on the root induction medium in a shaded greenhouse with 30% natural irradiance. Samples were then transferred to plastic pots containing a mixture of sterile sand and peat soil (1:2) at a planting density of 3 cm × 3 cm. The pots were incubated in a shaded greenhouse at 25 °C with 80–90% relative humidity. All plantlets survived.

## 3. Discussion

Wild *A. roxburghii* (Wall.) Lindl resources are endangered. Moreover, the low regeneration rate of commercial propagation methods has hindered the large-scale artificial cultivation of propagated plants [8]. This has restricted the commercial production of *A. roxburghii* [26] and kinsenoside. To develop an efficient method for the mass propagation of *A. roxburghii* and an alternative resource for kinsenoside production, we systematically installed an IPR-PLB protocol applicable for *A. roxburghii*. Additionally, we examined the kinsenoside and flavonoid contents during the induction and proliferation of PLBs.

### 3.1. Induction of A. roxburghii PLBs

Hormone treatments decreased the time required for PLBs to appear and increased the PLB induction rate (Table 1), which is consistent with the findings of an earlier study by Feng et al. [27]. These results reflect the importance of hormones for the formation of PLBs [10,14]. The PLB induction rate was higher on MS + 3 mg/L 6-BA + 0.5 mg/L NAA than that on MS medium lacking hormones (Table 1). The inclusion of 6-BA and NAA in the medium reportedly enhances the induction of *Oncidium* ’Sweet Sugar’ [28] and *Oncidium* Aloha ‘Iwanaga’ PLBs [29]. The ideal 6-BA and NAA concentrations are 0.5–20 mg/L and 0.1–1 mg/L, respectively [10,30,31]. These results indicate that 6-BA and NAA can increase the efficiency of orchid PLB induction [10,14]. The addition of ZT alone or with 2, 4-D substantially increased the PLB induction rate (Table 1). A similar result was obtained in previous studies on the induction of *Oncidium* sp. and *A. roxburghii* PLBs [10,15,16,32,33]. Accordingly, hormones are crucial factors for orchid PLB induction [10,14]. The PLB induction rate was highest (89 ± 2.08%) on MS + 3 mg/L 6-BA + 0.5 mg/L NAA + 0.8 mg/L ZT + 0.2 mg/L 2, 4-D. However, half-strength MS + 0.8 mg/L ZT + 0.2 mg/L NAA is the most suitable medium for inducing *Anoectochilus formosanus* Hayata PLBs [27]. In contrast, MS + 2.0 mg/L 6-BA + 0.2 mg/L 2, 4-D + 0.2 mg/L ZT was ideal for the induction of *A. roxburghii* PLBs [15]. Hence, the optimal medium for inducing *Anoectochilus* PLBs varies among species. This is in accordance with earlier findings, which confirmed that the optimal medium for many plant species, including orchids, depends on the germplasm [10,34]. Nodes from the middle of the stem had higher induction rates than the nodes near the bottom of the stem, and the two nodes near the apical shoot did not generate PLBs. The same results were reported for *A. roxburghii* originated in Guangxi, Taiwan, and Fujian [32]. Thus, selecting appropriate explants is critical for orchid PLB formation [10,14].

### 3.2. Proliferation of A. roxburghii PLBs

The PLBs differentiated to form plantlets during a 45-day cultivation with a 16 h light/8 h dark photoperiod (1500–2000 lux irradiation). However, secondary PLB masses were observed after intact or PLB sections were cultivated in darkness or under low-intensity light for 60 days, which implies darkness or weak light is essential for the proliferation of *A. roxburghii* PLBs. The importance of darkness or weak light for PLB induction was also observed for *Phalaenopsis* [10,34]. The time required for the formation of secondary PLB masses was unaffected by the use of intact PLBs or PLB sections. Han et al. reported a similar time required for the production of secondary PLBs from intact *A. roxburghii* PLBs [15]. The induction rate was higher for the PLB sections than for the intact PLBs, possibly because wounding or tissue damage promotes the generation of orchid PLBs [10]. The secondary PLB proliferation rate (in terms of the biomass) in this study (3.50 ± 0.1 times) was higher than that (2.150 times) previously reported for a small PLB mass (1 cm^3^) [16], implying that how explants are cut may also influence PLB proliferation.

### 3.3. Kinsenoside and Flavonoid Contents in PLBs

The observed changes in kinsenoside contents during the induction and proliferation of secondary PLBs were similar to the kinsenoside content profiles of *A. roxburghii* plants at different growth stages [35]. In this study, the peak kinsenoside content (34.27 ± 0.79 mg/g) during the proliferation of secondary PLBs was slightly lower than the kinsenoside content of the whole plant (38.68 ± 3.12 mg/g). Additionally, the PLBs proliferated extensively (3.50 ± 0.1 times; in terms of the biomass) in 1 month, which is similar to the rapid proliferation of *A. roxburghii* observed by Han et al. [16]. These findings suggest that proliferated PLBs may be useful for enhancing kinsenoside production. Isoquercitrin was exclusively tested in the leaf of *A. roxburghii* (Wall.) Lindl not in PLB, the possible reason being that PLB was induced from stem noses, where there is no isoquercitrin tested either.

Flavonol glycosides are bioactive compounds crucial for plant responses to various stresses, including UV radiation and temperature extremes [36,37,38,39]. Astragalin contents increased during the first 2 weeks of the cultivation. This suggests that wounding induced astragalin accumulation, whereas the PLB biomass increase was limited, during the first 2 weeks of cultivation. Conversely, the wound-induced accumulation of astragalin decreased, while the PLB biomass increased sharply, during the third and fourth weeks of cultivation, ultimately resulting in trace amounts of astragalin that were undetectable by HPLC.

Interestingly, *A. roxburghii* PLB masses can proliferate steadily for a stage [18], almost 2 years, which is conducive to the continuous large-scale propagation of *A. roxburghii* plants from proliferated PLBs and the sustained year-round production of kinsenoside.

### 3.4. Regeneration of PLBs: Shoot and Root Formation

Light is essential for *A. roxburghii* PLB regeneration. Earlier research revealed that light is important for the differentiation of PLBs into plantlets [7,8], similar to how darkness or weak light plays an important role in PLB induction [10,34]. The effects of hormones on IPR-PLB protocols for orchids have been investigated [10,14], which indicated that 6-BA and NAA are critical hormones for orchid propagation [10,14]. The highest shoot induction rate (10.5 shoots/PLB mass) was observed on MS + 3 mg/L 6-BA + 0.5 mg/L NAA (Table 2). However, the shoot length decreased with 6-BA concentrations increasing. Hence, low and high 6-BA concentrations promote the differentiation of shoots and inhibit shoot elongation and proliferation, respectively [40,41] (Table 2). This was also observed during the induction of *A. roxburghii* shoots from stem nodes [42]. Previous studies on *Cyrtopodium saintlegerianum* [43] and *Catasetum fimbriatum* [41] also confirmed this finding. The shoot induction rate for the IPR-PLB method was approximately 42 times, which was considerably higher than the *A. roxburghii* shoot regeneration rate when stem nodes were used (0.93–4 times) [7,44]. Shoots on MS + 0.5 mg/L NAA had the highest rooting rate (98.35 ± 0.05%) (Table 3). However, rooting was also observed for approximately 93% of the shoots on the MS + 0.5 mg/L NAA + 0.1 mg/L 6-BA as well as the MS + 0.5 mg/L NAA + 0.1 mg/L 6 − BA + 4 mg/L IBA. Therefore, NAA is vital for the induction of roots. Similar observations were reported for other orchid plants, such as *Dendrobium aqueum* [45] and *Cymbidium mastersii* [46]. The acclimation results demonstrated that a sterile sand and peat soil mixture (1:2) is appropriate for the regeneration of *A. roxburghii* plants from proliferated PLBs as well as from stem nodes [42] in a shaded greenhouse at 25 °C and 80–90% relative humidity.

## 4. Materials and Methods

### 4.1. Plant Materials

*Anoectochilus roxburghii* plants were cultivated in Baicaoyuan (latitude 30°15′, longitude 119°43′). Their identities were confirmed by Professor Runhuai Hu at Zhejiang A & F University. Uncracked capsules were collected and immersed in 75% ethanol for 1 min, surface-sterilized in 5% sodium hypochlorite for 40 min, and washed three or four times with sterile distilled water. The sterilized capsules were longitudinally divided in half to obtain seeds, which were sprinkled evenly onto solid MS medium in culture bottles, which were subsequently incubated for 3 months in a tissue culture room at 25 ± 2 °C with a 16 h light/8 h dark photoperiod (1500–2000 lux irradiation). The culture bottles were transferred to a greenhouse and incubated at 25 ± 2 °C under natural light conditions for another 3 months, until samples grew to a height of approximately 5–6 cm. The solid MS medium was supplemented with 30 g/L sucrose (pH 5.8) and solidified by the addition of 7 g/L agar. In accordance with national laws and regulations and the Convention on International Trade in Endangered Species of Wild Fauna and Flora (http://www.cites.org.cn/ accessed on 20 March 2022), no permits were required for the collection of *A. roxburghii* samples.

### 4.2. PLB Induction

The *A. roxburghii* plantlets were used for the PLB induction. After removing their leaves and roots, the stems were cut into segments with nodes of 0.3–0.5 cm in length. The stem nodes, which served as explants, were added to different PLB induction media (Table 1). For each tested medium, 30 Petri dishes were inoculated with 20 explants for the PLB induction at 25 ± 2 °C with a 16 h light/8 h dark photoperiod (1500–2000 lux irradiation). Fifteen Petri dishes per medium were randomly selected to record the time required for PLB emergence and to calculate the PLB induction rate at 30 dpi. The PLB induction rate (%) was calculated using the following formula: number of explants with PLBs/number of explants used to inoculate the medium × 100.

### 4.3. PLB Proliferation (Induction and Proliferation of Secondary PLBs)

The PLBs at 30–35 dpi were cut to prepare transverse sections (2–4 mm thick) for the inoculation of the optimized PLB induction medium, which was subsequently incubated at 25 ± 2 °C in darkness or with a 16 h light/8 h dark photoperiod (500–1000 lux irradiation) for the induction and proliferation of secondary PLBs. Thirty Petri dishes containing the optimized medium were each inoculated with 20 PLB sections. The cultured materials were transferred to new medium every 2 weeks. Fifteen Petri dishes were randomly selected to calculate the secondary PLB induction rate (%) at 60 dpi using the following equation: number of secondary PLB masses/number of PLBs used to inoculate the medium × 100.

Secondary PLB masses were cut into transverse sections for the analysis of PLB proliferation. All 30 Petri dishes were inoculated with 20 secondary PLB sections (approximately 0.5 g FW). Fifteen Petri dishes were randomly selected to calculate the secondary PLB proliferation rate in terms of the quantity and biomass at 30 dpi. Specifically, the secondary PLB proliferation rate (%) in terms of the quantity was calculated using the following formula: number of proliferated PLB masses/number of PLB masses used to inoculate the medium × 100. The secondary PLB proliferation rate (in terms of the biomass) was calculated using the following formula: weight of the proliferated PLBs/weight of the PLBs used for the inoculation.

### 4.4. Determination of Kinsenoside, Isoquercitrin, and Astragalin Contents

Fresh samples (0.5 g) of 3-month-old *A. roxburghii* plants cultivated in a greenhouse were homogenized in 5 mL 95% methanol at 4 °C. The samples were ultrasonicated for 50 min at 500 W and 40 kHz at room temperature. The resulting solution was centrifuged at 8000× *g* for 10 min at 4 °C and the supernatant was filtered through a 0.45 µm membrane. A 10 µL aliquot of the sample was analyzed using the HPLC–ELSD system. The kinsenoside standard (≥98%; HPLC grade) was supplied by Shanghai Yuanye Biotechnology Co., Ltd. (Shanghai, China). Its linear regression equation was *y* = 1992.5*x* + 39,737, where *x* represents the kinsenoside concentration and *y* represents the corresponding relative peak area. The correlation coefficient was 0.9996 and the linear range was 50–1600 µg/mL (Supplementary Figure S1). The HPLC system comprised the Waters™ 2695 HPLC pump separation module (Milford, MA, USA) and the Agilent NH2 column (250 mm × 4.6 mm, 5 μm; Agilent, Santa Clara, CA, USA). The mobile phase was acetonitrile and H_2_O (85:15, *v*/*v*). The flow rate was 1.0 mL/min and the column temperature was 30 °C. Signals were detected using the Waters 2424 ELSD, with a drift tube temperature of 80 °C and a gas flow rate corresponding to 40 psi (1 psi = 6.895 kPa). Each sample was analyzed in triplicate.

Fresh plant samples (0.5 g) were homogenized in 50 mL methanol at 4 °C. The samples were ultrasonicated for 40 min at 500 W and 40 kHz. After cooling, the samples were centrifuged at 8000× *g* for 10 min. The supernatant was filtered through a 0.45 µm membrane and then a 10 µL aliquot of the sample was used for the HPLC–PDA analysis. Isoquercitrin (≥98%; HPLC grade) and astragalin (≥99%; HPLC grade) standards were supplied by Shanghai Yuanye Biotechnology Co., Ltd. (Shanghai, China). The linear regression equations for the isoquercitrin and astragalin standards were *y* = 20,905*x* + 360,191 and *y* = 165,22*x* − 291,678, respectively, where *x* represents the standard concentration and *y* represents the corresponding relative peak area. The correlation coefficients for isoquercitrin and astragalin were 0.9969 and 0.9972, respectively, and the linear range for both standards was 13.28–425 µg/mL (Supplementary Figures S2 and S3). The HPLC system consisted of the Waters™ 2695 HPLC pump separation module (Milford), an autoinjector system (100 µL), and a Waters C18 column (250 mm × 4.6 mm, 5 μm; Agilent). The column temperature was 30 °C, the flow rate was 1 mL/min, and the injection volume was 20 μL. The mobile phase was acetonitrile (a) and 1% acetic acid (b). The gradient elution was as follows: 0–9 min, 20% (a) and 9–40 min, 20–38% (a). The detection wavelength was 360 nm.

### 4.5. Regeneration of PLBs: Shoot and Root Formation

The proliferated PLB masses were added to different media (Table 2) and then incubated in a tissue culture room at 25 ± 2 °C with a 16 h light/8 h dark photoperiod (1500–2000 lux irradiation) to induce shoot formation. Each medium was added to 30 tissue culture bottles, and each bottle was inoculated with 15 PLB masses. Fifteen bottles per medium were randomly selected to calculate the number of shoots. The shoot induction rate was calculated as follows: shoot induction rate = number of shoots/number of PLB masses used to inoculate the medium; total shoot induction rate = PLB induction rate × secondary PLB induction rate × secondary PLB proliferation rate × shoot induction rate.

Shoots (1.5–2 cm in height) were transferred to the rooting MS media (Table 3). Each medium was added to 25 bottles, and each bottle was inoculated with 12 shoots. At 30 dpi, 15 bottles per medium were randomly selected to determine the number of roots and the root length. The rooting rate was calculated as follows: number of plantlets/number of shoots used to inoculate the medium.

### 4.6. Acclimation of Plantlets

Plantlets with four leaves were cultivated in root-inducing medium in a tissue culture room at 25 ± 2 °C with a 16 h light/8 h dark photoperiod (1500–2000 lux irradiation) until well-developed roots were detected. The plantlets were acclimated under the optimized conditions established in our laboratory [42]. The plantlets were transferred to a shaded greenhouse with 30% natural irradiance and incubated for 15 days. The adherent medium was removed using tap water. The plantlets were then transferred to plastic pots containing a sterile sand and peat soil mixture (1:2), with a planting density of 3 cm × 3 cm. The plantlets were grown in a shaded greenhouse at 25 °C and 80–90% relative humidity. The survival rate was calculated after a 15-day incubation as follows: number of surviving plantlets/total number of plantlets.

### 4.7. Statistical Analysis

All experiments were performed using a randomized design. To analyze the generated data, the SPSS 25 statistical analysis software was used to perform the F-test and Q-test. The results are provided herein as the mean ± standard deviation.

## 5. Conclusions

In the present study, we assessed the utility of an IPR-PLB procedure for *A. roxburghii* to determine whether it is better than conventional propagation methods. The total conversion rate of shoots from proliferated PLB masses was much higher than that from stem nodes. The peak kinsenoside content of the proliferated PLB masses was slighter lower than that of the whole plant, but the proliferated PLB masses formed more quickly than the whole plant. Accordingly, the PLB masses may be used as explants for the large-scale propagation of *A. roxburghii* plants as well as for the commercial production of large amounts of kinsenoside. Isoquercitrin and astragalin accumulated in a tissue- or temporal-specific manner, and interpretation of the distribution of these two flavonoid monomers will facilitate their comprehensive exploitation and exploitation from *A. roxburghii* plants and PLBs.

## Figures and Tables

**Table 1:** Induction of PLBs on different MS medium (*n* = 300; data are presented as the mean ± standard deviation).

PGRs (mg/L)				PLB Appearance Time	PLB Induction Rate (%)
6-BA	NAA	ZT	2, 4-D		
0	0	0	0	25 ± 1.00	60 ± 1.50
3	0.5	0	0	20.3 ± 1.53	65 ± 1.44
3	0.5	0.2	0	22.6 ± 2.08 *	80 ± 1.52
3	0.5	0.5	0	23.6 ± 1.52	83.5 ± 1.32 *
3	0.5	0.8	0	21.0 ± 1.00	83 ± 1.22
3	0.5	0.8	0.2	19.3 ± 1.53 *	89 ± 2.08 *

Note: The asterisk indicates a significant difference (*p* < 0.05) between the MS with hormones and the MS without hormones.

**Table 2:** Shoot induction from secondary PLBs (*n* = 225; data are presented as the mean ± standard deviation).

Hormone (mg/L)		Induction Rate (Shoots/PLB Mass) (Mean ± SD)	Shoot Length (Mean ± SD) (cm)	Shoot Diameter (Mean ± SD) (mm)
6-BA	NAA			
0	0	5.0 ± 0.23	3.01 ± 0.45	2.02 ± 0.44
1.0	0.5	6.5 ± 0.51 *	2.89 ± 0.56	2.61 ± 0.11
2.0	0.5	8.2 ± 0.05	2.78. ± 0.11 *	2.97 ± 0.45
3.0	0.5	10.5 ± 0.64 *	2.79 ± 0.75 *	3.19 ± 0.33 *
4.0	0.5	9.1 ± 0.67	2.56 ± 0.33	2.60 ± 0.22
5.0	0.5	4.3 ± 1.2 *	2.43 ± 0.24	2.43 ± 0.35

Note: The asterisk indicates a significant difference (*p* < 0.05) between the MS medium with hormones and the MS medium without hormones.

**Table 3:** Rooting of *A. roxburghii* plantlets (*n* = 180; data are presented as the mean ± standard deviation).

PGRs (mg/L)			Rooting Rate (Mean ± SD)%	Root NUMBER (Mean ± SD) cm	Root Length (Mean ± SD) cm
NAA	6-BA	Indole Butyric Acid (IBA)			
0.5	0	0	98.35 ± 0.05	3.22 ± 0.21	3.51 ± 0.12
0.5	0.1	0	93.45 ± 0.51 *	2.89 ± 0.56	3.34 ± 0.17 *
0.5	0.1	4	93.14 ± 0.23 *	3.01 ± 0.45 *	2.89 ± 0.31

Note: The asterisk indicates a significant difference (*p* < 0.05) between the rooting medium and the MS medium supplemented with only 0.5 mg/L NAA.

## Data Availability

Not applicable.

## Supplementary Materials

The following supporting information can be downloaded at: www.mdpi.com/xxx/s1-s3, Figure S1: The linear regression equation of kinsenoside. Kinsenoside concentration is the *x* axis and the corresponding relative peak area is the *y* axis. The linear regression equation was *y* = 1992.5*x* + 39,737, the correlation coefficient is 0.9996, and the linear range is 50–1600 µg/mL; Figure S2. The linear regression equations of isoquercitrin. Its concentration is the *x* axis and the corresponding relative peak area is the *y* axis. The linear regression equations was *y* = 20,905*x* − 360,191, the correlation coefficients is 0.9969, and the linear range for the equation is 13.28–425 µg/mL; Figure S3. The linear regression equations of astragalin. Its concentration is the *x* axis and the corresponding relative peak area is the *y* axis. The linear regression equations was *y* = 16,522*x* − 291,678, the correlation coefficients is 0.9972, and the linear range for both equations is 13.28–425 µg/mL.

## Abbreviations

Days post-inoculation: dpi; protocorm-like body: PLB; 6-benzylaminopurine: 6-BA; naphthaleneacetic acid: NAA; 2,4-dichlorophenoxyacetic acid: 2, 4-D; zeatin: ZT; indole butyric acid: IBA; fresh weight: FW; evaporative light-scattering detector: ELSD.
