# Supplementary material for: Induction, Proliferation, Regeneration and Kinsenoside and Flavonoid Content Analysis of the Anoectochilus roxburghii (Wall.) Lindl Protocorm-like Body
Source: Plants (Basel). 2022 Sep 21;11(19):2465. doi: 10.3390/plants11192465 (PMC9570929; doi:10.3390/plants11192465)
Supplement: Supplementary file 1 [file plants-11-02465-s001.zip › plants-1776084-supplementary.pdf]

plants MDPI

Supplementary material

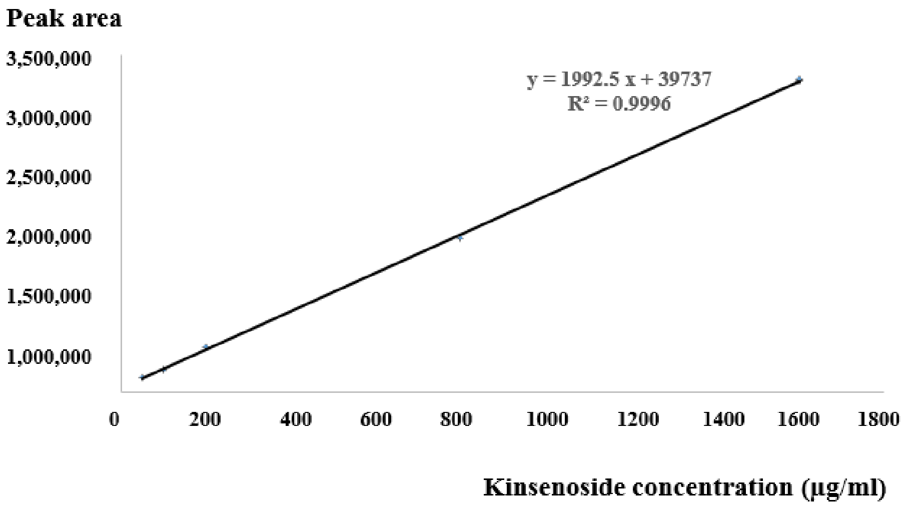

Figure S1. The linear regression equation of kinsenoside. Kinsenoside concentration is the x axis and the corresponding relative peak area is the y axis. The linear regression equation was y = 1992.5 x + 39737, the correlation coefficient is 0.9996, and the linear range is 50–1600 μg/mL.

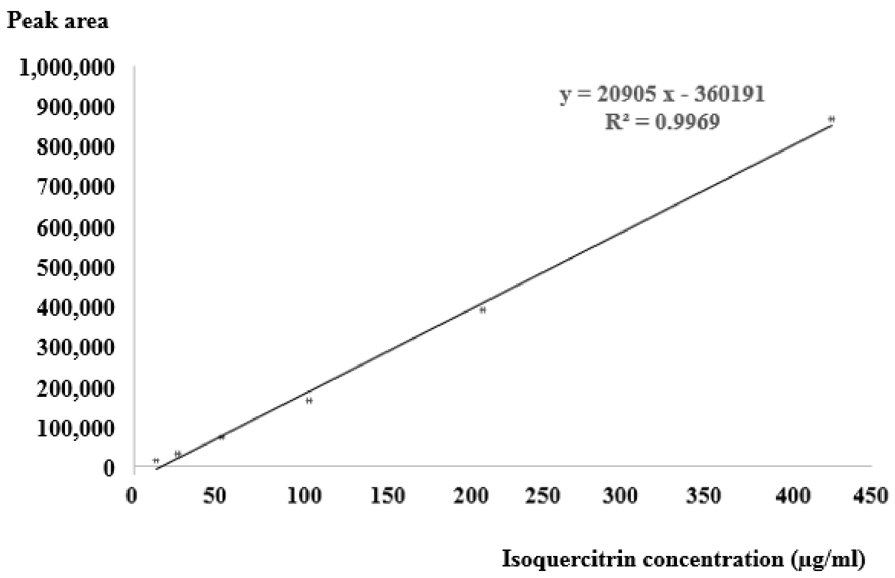

Figure S2. The linear regression equations of isoquercitrin. Its concentration is the x axis and the corresponding relative peak area is the y axis. The linear regression equations was y = 20905 x - 360191, the correlation coefficients is 0.9969, and the linear range for the equation is 13.28–425 μg/mL.

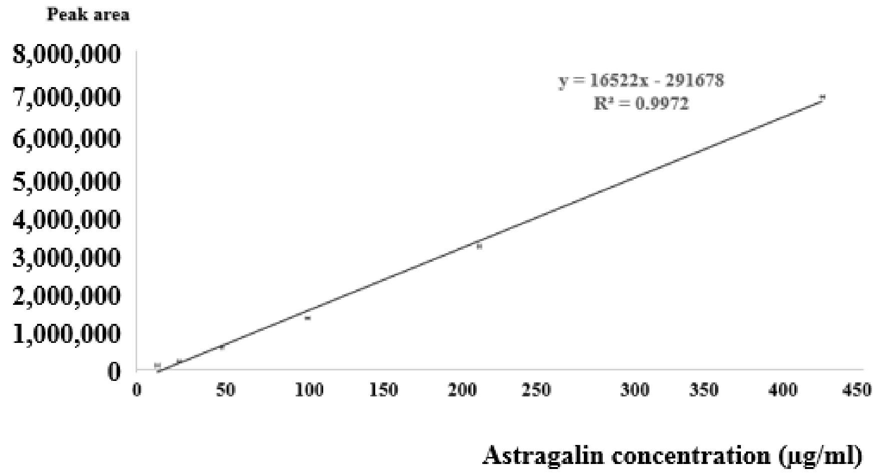

Figure S3. The linear regression equations of astragalin. Its concentration is the x axis and the corresponding relative peak area is the y axis. The linear regression equations was y = 16522 x–291678, the correlation coefficients is 0.9972, and the linear range for both equations is 13.28–425 μg/mL.
